# Can Conditioning Activity with Blood Flow Restriction Impact Neuromuscular Performance and Perceptual Responses to Exercise?

**DOI:** 10.3390/sports13080243

**Published:** 2025-07-24

**Authors:** Robson Conceição Silva, Leandro Lima Sousa, Hugo de Luca Correa, Thailson Fernandes Silva, Lucas de Souza Martins, Pedro Felix, Martim Bottaro, Denis César Leite Vieira, Carlos Ernesto

**Affiliations:** 1 Graduate Program of Physical Education, Catholic University of Brasilia, Taguatinga–DF, Brasilia 71966-700, Brazilcarlosf@p.ucb.br (C.E.); 2 College of Physical Education, University of Brasilia–UnB, Asa Norte–DF, Brasília 700910-900, Brazil; 3 INSERM UMR1093-CAPS & Centre d’Expertise de la Performance, Université Bourgogne Europe, UFR des Sciences du Sport, 21000 Dijon, France

**Keywords:** PAP, PAPE, vascular occlusion, resistance training, acute exercise effect, random crossover design, isokinetic test

## Abstract

Low-load conditioning activity with blood flow restriction has been addressed as an efficient method to enhance an individual’s performance during their main exercise activity. However, the optimal degree of blood flow restriction remains unclear. Therefore, this study investigated the acute effects of low-load conditioning activity with different degrees of blood flow restriction on muscle strength, power, and perceived exertion. Twenty recreationally trained men (20.9 ± 2.3 years) participated in a randomized crossover design including three conditions: control, low-load blood flow restriction at 50%, and 75% of total arterial occlusion pressure. Participants performed squats (three sets of ten reps) followed by isokinetic assessments of the knee flexor and extensor performance at 7 and 10-min post-exercise. The session rating of perceived exertion (SRPE) was recorded 30 min after each session. No significant effects were observed for condition, time, or their interaction on peak torque, total work, or average power (*p* < 0.05). However, SRPE was significantly higher in the 75% BFR condition compared to both the 50% BFR and control conditions (*p* < 0.05), with no difference between the 50% BFR and control. These findings suggest that low-load conditioning activity with blood flow restriction does not acutely enhance neuromuscular performance. However, a higher degree of restriction increases perceived exertion.

## 1. Introduction

Improving muscle strength and power is a key adaptation for athletic performance in many sports, particularly those involving running, jumping, throwing, and kicking [1]. Accordingly, coaches and athletes often implement strategies to acutely enhance these physical attributes and thereby improve performance during the main exercise activity [2,3,4,5]. Indeed, several studies have shown that both maximal and submaximal muscle contractions can acutely enhance performance in subsequent tasks [6,7,8,9]. In this context, performing high-load conditioning activities (typically ranging from 85 to 90% of one-repetition maximum) has been shown to induce temporary increases in muscle strength and power after 7 to 8 min of rest [10]. Therefore, pre-activation through high-load conditioning, followed by passive recovery, may enhance subsequent performance [11].

However, high loads are often associated with an increased perception of effort [12,13], which can make such protocols uncomfortable and limit their practicality. As increases in muscle water content and the recruitment of fast-twitch muscle fibers are believed to contribute to performance improvements following conditioning activities [2], alternative methods such as low-load conditioning activity with blood flow restriction (BFR) have been proposed. BFR exercise has been suggested to increase the accumulation of muscle metabolites, which may promote fluid shifts into the muscle and stimulate fast-twitch fiber recruitment [14,15,16]. Indeed, a previous study reported that lunge exercise with blood flow restriction positively affected drop jump performance [17]. However, that study determined the occlusion pressure based on resting systolic blood pressure, a method that may pose more safety concerns compared to individualized total arterial occlusion pressure measured via Doppler ultrasound [18].

The degree of blood flow restriction has been reported inconsistently in the literature, particularly regarding its impact on muscle swelling and water content after exercise [19,20]. Moreover, higher levels of restriction may affect perceived effort and, consequently, influence fatigue [19]. Thus, it is reasonable to suggest that low-load conditioning activity with blood flow restriction may serve as an effective strategy to enhance muscle strength and power and, thereby, an individual’s performance during their main exercise activity. Nonetheless, the effects of applying different blood-flow restriction intensities, specifically using the safer Doppler-based arterial occlusion method, have not yet been systematically tested. Therefore, the present study aimed to investigate whether a low-load conditioning activity with blood flow restriction can improve muscle strength and power and whether the degree of restriction affects these adaptations. In addition, we examined session ratings of perceived exertion in low-load conditioning activities with different levels of blood flow restriction. Based on previous reports, which hypothesized that low-load BFR conditioning could enhance neuromuscular performance regardless of the degree of restriction, we anticipated that higher restriction levels would result in greater perceived exertion.

## 2. Materials and Methods

### 2.1. Participants

Twenty recreationally trained men participated in this study. The mean age ± standard deviation (SD), height, body mass, body fat, and systolic and diastolic blood pressure were 20.9 ± 2.3 years, 1.75 ± 0.06 m, 74.5 ± 11.1 kg, 10.2 ± 5.6%, 123.4 ± 5.19 mmHg, and 67.6 ± 4.36, respectively. None of the participants reported any lower limb injuries or back pain in the last three months, any specific hamstring or triceps surae injuries in the last two years, nor the use of medicine and nutritional supplements that could have an impact on blood pressure. Additionally, exclusion criteria also included resting blood pressure ≥ 140 × 90 mmHg, existing heart disease, peripheral vascular disease, diabetes, BMI ≥ 30, and one or more risk factors for thromboembolism [21]. The participants were asked to maintain their regular physical activities and dietary intake. They were also advised to refrain from intensive activity for at least two days before an experimental session. All volunteers were fully informed about the experimental procedure and purpose of the study. They read and signed an informed consent form. This study was approved by the institutional review board (CAAE 39652920.4.0000.0029) and was conducted in accordance with the Declaration of Helsinki. The sample size was calculated a priori using G*Power (version 3.1.9.6) considering a Partial Etta Square (ƞp^2^) of 0.15, an effect size of 0.42, a power of 0.8, and a probability error of 0.05. Thus, a sample of 20 volunteers was deemed sufficient.

### 2.2. Experimental Procedures

**Figure 1:**
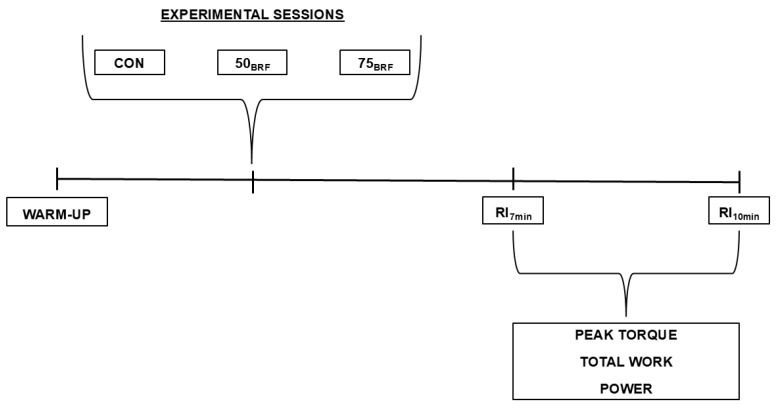
Experimental design. CON = control session; 50_BFR_ = 50% blood flow restriction session; 75_BFR_ = 75% blood flow restriction session; RI_7min_ = rest interval of 7 min; RI_10min_ = rest interval of 10 min.

This was a randomized crossover study. Each participant completed four visits to the laboratory, including one for familiarization and three for experimental and control sessions (Figure 1). All sessions were separated by at least 48 h. Additionally, all testing sessions were conducted at the same time of day to reduce the influence of circadian rhythms, and participants were instructed not to consume stimulant (caffeine) or depressant (alcohol) substances 24 h prior to each session.

During the familiarization session, the participants completed the Physical Activity Readiness Questionnaire (PAR-Q), International Physical Activity Questionnaire (IPAQ-Short Version), and Assessment of Thrombosis Risk Factors (ATRF) questionnaires. In addition, measurements of the ankle-brachial index (ABI) and anthropometric measures were taken [22,23]. Following this, the order of legs to be evaluated in the bilateral knee flexion and extension isokinetic test was determined by random selection, and the same order was maintained for all sessions. Additionally, body mass was assessed (Toledo 2096PP, São Paulo, Brazil) alongside height (Country Technology, Gays Mills, WI, USA) and a body composition assessment using a dual-energy X-ray absorptiometry (DXA) densitometer (Lunar DPX-IQ, Madison, WI, USA).

In each session, participants initially sat and rested for 5 min to measure blood pressure. Then, they performed a standardized warm-up, which consisted of 5 min of cycling on an ergometer (Monark 894E Peak Bike, Monark Exercise AB, Vansbro, Sweden) at a constant power output of 1 W/kg of body mass [24]. Then, after a three-minute rest interval, total arterial occlusion pressure was measured in the experimental sessions. The isokinetic test for the assessment of muscle strength and power was performed 7 and 10 min after the experimental protocol as the optimal times to verify post-activation performance enhancements from conditioning activities [10]. In the control session, only the warm-up and the isokinetic test were performed.

### 2.3. Total Arterial Occlusion Protocol

The total arterial occlusion pressure of the participants was measured in an upright position, with their arms crossed and hands on their shoulders as they performed the conditioning activity low-load and blood flow restriction protocol. In this regard, inflatable cuffs (20 cm × 42 cm, Premium, Shangai, China) were used, which controlled the pressure through gauges with an accuracy of 2 mmHg (Premium, Shangai, China) positioned on the proximal part of the thigh (as close as possible to the inguinal fold) [18]. The participants rested with the knees extended and the portable vascular Doppler probe (DV 610B, Medmega, Franca, SP, Brazil) positioned over the dorsalis pedis artery using conducting gel; the cuff was inflated until the blood flow completely ceased. The value of the total arterial occlusion pressure was recorded, and the pressure to be used was calculated depending on the randomized protocol.

### 2.4. Experimental Sessions

The load conditioning activity with blood flow restriction protocols consisted of performing squats with a range of motion of 90 degrees. Three sets of ten repetitions were performed with a cadence of 1 s for the concentric phase and 1 s for the eccentric phase, controlled using a digital metronome (Model DM50, Seiko Sports Life Co., Ltd., Shangai, China), with a 2 min rest interval between sets, totaling 5 min of low-load conditioning activities with blood flow restriction. The blood flow restriction was 50% or 75% of the total arterial occlusion pressure, according to the experimental session. During the control session, the individuals rested in the seated position for 7 min, waiting to begin the isokinetic test procedures.

### 2.5. Neuromuscular Performance

The neuromuscular performance was assessed using an isokinetic dynamometer and the Biodex System III isokinetic dynamometer (Biodex Medical, Inc., Shirley, NY, USA) for the following variables: peak torque, total work, and power (Biodex Medical, Inc., Shirley, NY, USA). All assessments were performed at 180°/s with 3 maximal repetitions of knee extension and flexion [25]. The first leg was assessed 7 min after the conditioning activity, while the second leg was assessed after 10 min [10]. The participants were positioned comfortably in a dynamometer chair and secured with belts around the trunk, pelvis, and thigh regions to minimize extraneous body movements that could affect the test [26]. The lateral epicondyle of the femur was used as a marker to align the knee’s axis of rotation with the equipment’s axis of rotation, allowing for an unrestricted and comfortable flexion and extension movement of the knee from a position of 90 degrees flexion to full extension (range of motion of 70 degrees). During the test, the participant received verbal encouragement and visual feedback [27,28,29]. Furthermore, the all-testing procedures were conducted by the same investigator.

### 2.6. Session Rating Perceived Exertion

The session rating of perceived exertion (SRPE) was assessed using the Borg CR-10 scale for 30 min after the sessions to avoid any interference from the exercises and testing efforts. The individuals were instructed to report their perceived exertion considering all procedures during an experimental session in the Borg CR-10, with 0 representing rest and 10 representing the maximal effort. All participants received the same instructions, and all procedures were performed by the same investigator [30,31].

### 2.7. Statistical Analysis

Data were presented as the mean ± standard deviation (SD). The normality of the data was assessed using the Shapiro–Wilk test. A two-way (condition × time) repeated measures analysis of variance was used to assess the peak torque, total work, and power. These conditions correspond to control sessions and low-load conditioning activities with a degree of blood flow restriction of 50% or 75%. The times used were 7 min and 10 min. Moreover, a Friedman analysis of variance was used for session perceived exertion. The Bonferroni post hoc tests were performed in the case of a significant effect or interactions. The level of significance for all comparisons was set at *p* < 0.05. All statistical procedures were performed in JASP (version 0.14, JASP Team 2020, University of Amsterdam, available free at http://jasp-stats.org/download (accessed on 10 July 2025)).

## 3. Results

There was no effect on time (*p* = 0.246; F = 1.432; ηρ^2^ = 0.070, and *p* = 0.452; F = 0.698; ηρ^2^ = 0.030), condition (*p* = 0.939; F = 0.063; ηρ^2^ = 0.003, and *p* = 0.495; F = 2.357; ηρ^2^ = 0.036), or the condition × time interaction (*p* = 0.291; F = 1.239; ηρ^2^ = 0.061, and *p* = 0.949; F = 1.192; ηρ^2^ = 0.001) for the peak torque of the knee extensors and flexors, respectively. Similarly, there was no effect on time (*p* = 0.391; F = 0.771; ηρ^2^ = 0.039, and *p* = 0.541; F = 0.388; ηρ^2^ = 0.020), condition (*p* = 0.455; F = 0.804; ηρ^2^ = 0.041, and *p* = 0.299; F = 1.245; ηρ^2^ = 0.062), or the condition × time interaction (*p* = 0.194; F = 1.713; ηρ^2^ = 0.083, and *p* = 0.630; F = 0.467; ηρ^2^ = 0.024) for the total work of the knee extensors and flexors, respectively. Additionally, there was no effect on time (*p* = 0.278; F = 1.249; ηρ^2^ = 0.062, and *p* = 0.414; F = 0.698; ηρ^2^ = 0.035), condition (*p* = 0.139; F = 2.080; ηρ^2^ = 0.099, and *p* = 0.108; F = 2.357; ηρ^2^ = 0.110), or the condition × time interaction (*p* = 0.491; F = 0.724; ηρ^2^ = 0.037, and *p* = 0.307; F = 1.192; ηρ^2^ = 0.059) for the average power of the knee extensors and flexors, respectively (Table 1). Session perceived exertion had a significant effect (*p* = 0.001; X^2^_F_ = 25.795) with greater perceived exertion in the low-load conditioning activity with a high degree of blood flow restriction compared to a low degree of restriction (*p* = 0.001) and the control session (*p* = 0.001). Additionally, there was a significant difference between the low-load conditioning activity with a low degree of blood flow restriction and the control session (*p* = 0.004; Table 2).

## 4. Discussion

This study verifies the acute effects of low-load conditioning activity with different degrees of blood flow restriction on performance and perceived exertion. The results indicate that low-load conditioning activity with blood flow restriction does not acutely enhance muscle strength and power independently of the degree of restriction. Moreover, sessions with low-load conditioning activity with blood flow restriction induce greater perceived exertion compared to the control session, regardless of the restriction level. However, a higher degree of restriction elicits significantly greater perceived exertion than lower degrees of exertion during the blood flow restriction condition.

A previous study reported greater improvements in jump performance after low-load conditioning activity with blood flow restriction compared to a control session [17]. Indeed, blood flow restriction is suggested to be an efficient method to enhance performance in subsequent activities [32]. As such, it may induce the recruitment of fast-twitch muscle fibers [16], muscle swelling, and increase the muscle water content [14,33,34,35,36], which, in turn, is associated with improvements in an individual’s performance during their main exercise activity [2]. Conversely, our study suggests that there is no effect on the low-load conditioning activity with blood flow restriction regarding muscle strength and power performance.

The absence of performance enhancement observed in our study may be explained by methodological factors, particularly the cuff pressure used to apply the blood flow restriction [17,18]. A previous study reported improvements in muscle performance following low-load conditioning activity with BFR using a cuff pressure set at 130% of resting systolic blood pressure [17]. In contrast, our study used individualized cuff pressures set at 50–75% of total arterial occlusion pressure, which corresponds to lower absolute pressures compared to 130% of systolic blood pressure (e.g., 139 mmHg vs. 158 mmHg, respectively). Although the use of Doppler-based total arterial occlusion is considered a safer and more standardized method for determining BFR pressure [18], it may not be the most effective approach for inducing performance enhancement in the context of pre-activation. This highlights a potential trade-off between safety and efficacy in BFR applications during conditioning activities.

Our study also found that session-perceived exertion was greater following low-load conditioning activity performed with a high degree of blood flow restriction compared to a low degree. Similarly, a previous study reported more negative affective responses and greater fatigue during resistance exercise with 80% BFR compared to 40% BFR [19]. The increased perception of discomfort and reduced enjoyment associated with exercise with higher blood flow restriction levels may be attributed to lower muscle oxygen availability, which leads to greater metabolic stress and, consequently, a more uncomfortable exercise experience [37,38,39]. Furthermore, a higher perception of muscle activation has been reported during resistance exercises with greater blood flow restriction intensity, which may further contribute to the sensation of discomfort [19]. These factors may help explain our finding of higher perceived exertion in the high- versus low-restriction blood flow restriction conditions during low-load conditioning activity. Thus, considering that affective responses such as perceived effort might influence adherence and are also recognized as important indicators of fatigue [19,40,41,42], they should be taken into account in practical applications within both clinical and sports settings. However, if the primary objective is solely to enhance neuromuscular performance, perceptual responses may not represent a limiting factor. The study has some limitations that should be mentioned. One limitation concerns the method used to determine the degree of blood flow restriction. Although using Doppler-based total arterial occlusion is considered the safest approach [18], a previous study reported improvements in performance following low-load conditioning activity with blood flow restriction when the cuff pressure was based on resting systolic blood pressure [17]. Furthermore, the Doppler device is not widely accessible in practical or athletic settings, which limits its applicability. Therefore, despite being the safest method, it may not be the most feasible or effective for enhancing performance in real-world scenarios. Additionally, although participants underwent a familiarization session, the potential influence of a learning effect on isokinetic performance cannot be entirely ruled out and should be considered another limitation of this study.

## 5. Conclusions

The present study showed no effect of low-load conditioning activity with blood flow restriction on muscular performance, regardless of the degree of restriction. However, performing a low-load conditioning activity with a higher degree of blood flow restriction resulted in greater perceived effort during the exercise session compared to both the control condition and the low-restriction condition. From a practical standpoint, our findings suggest that low-load conditioning activities with blood flow restriction should not be recommended for enhancing an individual’s performance during exercise activity, as it does not improve muscle strength or power and increases perceived exertion. However, this interpretation should be made with caution, as the use of high-degree blood flow restriction based on Doppler-derived total arterial occlusion, although considered safe, might not be the most practical or effective approach for performance enhancement in real-world settings.

## Figures and Tables

**Table 1:** Neuromuscular performance.

	Session		Peak Torque (Nm)	Total Work (J)	Power (W)
Knee extensors	CON	RI_7min_	175.11 ± 26.55 (162.69–187.53)	412.68 ± 78.70 (375.85–449.51)	291.63 ± 59.70 (263.69–319.57)
		RI_10min_	176.99 ± 27.92 (163.93–190.06)	413.35 ± 82.76 (374.62–452.08)	301.28 ± 63.28 (271.66–330.90)
	50_BRF_	RI_7min_	173.33 ± 28.12 (186.49–160.17)	413.55 ± 72.23 (379.74–447.35)	297.89 ± 58.14 (270.67–325.10)
		RI_10min_	178.73 ± 29.00 (165.16–192.30)	423.62 ± 76.00 (388.05–459.19)	302.93 ± 59.97 (274.87–330.99)
	75_BRF_	RI_7min_	173.99 ± 27.12 (161.30–186.68)	413.28 ± 74.87 (378.24–448.31)	300.80 ± 56.49 (274.36–327.24)
		RI_10min_	179.29 ± 24.93 (167.62–190.58)	430.42 ± 62.80 (401.02–459.81)	313.29 ± 52.85 (268.55–338.03)
Knee flexors	CON	RI_7min_	97.22 ± 16.02 (89.72–104.72)	242.67 ± 46.54 (220.88–264.45)	168.47 ± 30.56 (154.17–182.77)
		RI_10min_	96.11 ± 15.95 (88.65–103.58)	237.07 ± 49.28 (214.00–260.13)	161.96 ± 34.13 (152.38–182.29)
	50_BFR_	RI_7min_	96.73 ± 17.03 (88.76–104.70)	245.10 ± 47.51 (222.86–267.33)	167.34 ± 31.96 (157.27–188.36)
		RI_10min_	95.51 ± 18.55 (86.83–104.19)	242.44 ± 53.15 (217.56–267.31)	164.11 ± 40.11 (145.99–177.94)
	75_BFR_	RI_7min_	98.49 ± 17.48 (90.31–106.66)	246.82 ± 47.94 (224.38–269.25)	172.82 ± 33.21 (145.34–182.88)
		RI_10min_	97.05 ± 17.80 (88.72–105.38)	245.41 ± 51.65 (221.27–269.58)	172.99 ± 38.34 (155.04–190.93)

Data are reported in mean ± standard deviation (95% confidence intervals); Nm = Newtons × Meters; J = Joules; W = Watts; CON = control session; 50_BFR_ = 50% blood flow restriction session; 75_BFR_ = 75% blood flow restriction session; RI_7min_ = rest interval of 7 min; RI_10min_ = rest interval of 10 min.

**Table 2:** Session rating perceived exertion.

	SRPE
CON	1.4 ± 0.5 * (1.2–1.6)
50_BFR_	2.0 ± 0.8 *^#^ (1.6–2.4)
75_BFR_	3.3 ± 1.0 ^#^ (2.8–3.7)

Data are reported in mean ± standard deviation (95% confidence intervals); SRPE = session rating perceived exertion; CON = control session; 50_BFR_ = 50% blood flow restriction session; 75_BFR_ = 75% blood flow restriction session; RI_7min_ = recovery interval of 7 min; RI_10min_ = recovery interval of 10 min. * Less than 75_BFR_ (*p* < 0.05); ^#^ greater than CON (*p* < 0.05).

## Data Availability

The original data presented in this study are openly available at Zenodo: https://doi.org/10.5281/zenodo.15755621.
